# Impact of Obesity on In-Hospital Morbidity and Mortality Among Patients Admitted for Acute Exacerbations of Chronic Obstructive Pulmonary Disease (COPD)

**DOI:** 10.7759/cureus.35138

**Published:** 2023-02-18

**Authors:** Poorva Bhide, Jay Bapaye, Gaurav Mohan, Medha Ghose, Jayashree Ravilla, Siva Naga S Yarrarapu, Doantrang Du

**Affiliations:** 1 Internal Medicine, Monmouth Medical Center, Long Branch, USA; 2 Internal Medicine, Rochester Regional Health, Rochester, USA; 3 Internal Medicine, RWJBarnabas Health, Long Branch, USA

**Keywords:** hospital charges, tracheostomy, length of stay, mortality, morbidity, non-invasive ventilation, mechanical ventilation, obesity, chronic obstructive pulmonary disease, copd

## Abstract

Background

Obesity has been considered to be a risk factor for increased morbidity and mortality among patients with cardiopulmonary diseases. The burden of chronic obstructive pulmonary disease (COPD) and obesity is very high in the United States. We aimed to use the National Inpatient Sample (NIS) to evaluate the impact of obesity on the outcomes of patients hospitalized with COPD exacerbation.

Materials & Methods

This is a retrospective cohort study from the NIS database involving adult patients hospitalized for COPD exacerbation in the year 2019 obtained using the international classification of diseases, 10^th^ revision coding system (ICD-10). Obese and morbidly obese subgroups were identified. Statistical analyses were done using the Stata software, and regression analysis was performed to calculate odds ratios. Adjusted odds ratios (aOR) were calculated after adjusting for potential confounders.

Results

Among patients hospitalized for COPD exacerbations, mortality rates were lower among obese and morbidly obese patients; aOR 0.72 [0.65, 0.80] and aOR 0.88 [0.77-0.99], respectively. Obese and morbidly obese were more likely to require non-invasive ventilation aOR 1.63 [1.55, 1.7] and aOR 1.93 [1.85-2.05], respectively, and were more likely to require mechanical ventilation aOR 1.25 [1.19, 1.31], and aOR 1.53 [1.44-1.62], respectively. The tracheostomy rate was 1.17%, 0.83%, and 0.38% among patients with morbid obesity, obesity, and nonobese patients, respectively. Obese (aOR 1.11 [1.07-1.14]) and morbidly obese patients (aOR 1.21 [1.16-1.26]) had higher odds of being discharged on home oxygen and to a skilled nursing facility (SNF), aOR 1.32[1.27-1.38] and aOR 1.37 [1.3-1.43], respectively. Average hospital charges and length of hospitalization were significantly higher for morbidly obese and obese patients as compared to non-obese patients (p < 0.01).

Conclusions

Among admissions for COPD exacerbation, the rates of non-invasive ventilation, mechanical ventilation, tracheostomy, discharge with supplemental oxygen, length of hospitalization, hospitalization charges, and discharge to an SNF were higher among obese patients representing a higher morbidity and healthcare utilization in this group. This, however, did not translate into increased mortality among obese patients admitted with COPD exacerbations, and further randomized controlled trials are required to confirm our findings.

## Introduction

Obesity is a growing cause of concern in the United States (US) in the past few decades. The annual healthcare cost attributable to obesity exceeds $700 billion each year worldwide, with an annual economic burden of about $100 billion in the US [1]. Obesity affects outcomes in several cardiopulmonary disease processes, especially among critically ill patients [2-4]. Obesity has a significant impact on the pulmonary system, as it is directly involved in the pathophysiology of obesity hypoventilation syndrome and obstructive sleep apnea [5,6].

Chronic obstructive pulmonary disease (COPD) is a leading cause of death, with a significant financial burden on healthcare resources in the United States [7]. Up to 65% of the COPD population is overweight (Body mass index (BMI) 25-29.99) or obese (BMI>30) [8]. Although the impact of obesity on all-cause mortality and morbidity cardiovascular diseases is established, a protective association has been noted in the effect of obesity on COPD patients [9]. The impact of obesity on respiratory pathophysiology and symptom intensity in patients with COPD is not well known. It is hypothesized that patients with COPD who are obese may have some favorable lung mechanics like limited hyperinflation of the lungs, and this may be, in part, the reason that a negative association has been noted between obesity and mortality among them [10]. In addition to mortality, COPD is also associated with morbidity in the form of hospitalization for non-invasive ventilation, oxygen dependence, the requirement of intubation/tracheostomy, and nursing home stay. Our study aims to explore the impact of obesity on the disease severity, in-hospital outcomes, morbidity, and mortality of patients admitted for acute exacerbation of COPD (AECOPD).

## Materials and methods

Design and data source

This study is a retrospective cohort study involving adult patients (aged>18) hospitalized for exacerbation of chronic obstructive pulmonary disease (COPD) in the US between January 1, 2019, and December 31, 2019. Data were obtained from the Nationwide Inpatient Sample (NIS) database for 2019. The NIS is the largest publicly available database, covering more than 98% of the US population and approximately 1,000 hospitals. It approximates discharges from US community hospitals, excluding rehabilitation and long-term acute hospitals. The database uses the international classification of diseases, 10^th^ revision, and clinical modification/procedure coding system (ICD-10-CM/PCS) [11].

Study population

This study included adult patients with a primary discharge diagnosis of acute exacerbation of COPD or a primary diagnosis of acute respiratory failure with a secondary diagnosis of COPD. Obese patients were identified based on the presence of a secondary discharge diagnosis of obesity. Patients were excluded if their age was less than 18 years. Sub-group analysis was done on morbidly obese patients (body mass index >40). Patient characteristics and comorbidities in all groups were recorded. 

Outcome measures

The primary outcome was comparing the mortality; and the incidence of non-invasive (NIV) and mechanical ventilation (MV) or endotracheal intubation as a measure of clinical severity among patients admitted for COPD exacerbation depending on the presence or absence of obesity. Secondary outcomes included length of stay and hospital charges accrued as a measure of healthcare utilization cost among both groups, supplemental oxygen on discharge, incidence of tracheostomy and discharge to a skilled nursing facility (SNF) as a measure of morbidity status, and the incidence of cardiac arrest and septic shock as a measure of complications during the hospital stay. 

Statistical analysis

Data were analyzed using Stata® (Statistics and Data) Version 17 BE software (StataCorp, Texas, USA). All analyses were conducted using the weighted samples for national estimates in adjunct to Healthcare Cost and Utilization Project (HCUP) regulations for using the NIS database. Co-morbidities were calculated as proportions of the cohort, and the Chi-squared test was used to compare the association between the non-obese and the obese subgroups. Multivariate regression analysis was done to adjust for possible confounders while calculating the primary and secondary outcomes. The patient's comorbidities were obtained during the literature review. A univariate screen was done to confirm these factors further. A p-value of 0.05 was set as the threshold for statistical significance in the regression analysis. Odds ratios were calculated for all outcomes and were adjusted for age, sex, race, insurance status, and Charleston comorbidity index (CCI).

Ethical considerations

The NIS database does not contain any patient identifiers. Since 2012, the NIS has also removed state-level and hospital identifiers. This is in accordance with HIPAA regulations and respects patient protection and anonymity. All NIS-based studies are thus exempt from institutional review board approval. 

## Results

Baseline characteristics of the study population

A total of 674,080 weighted hospitalizations for COPD exacerbation were included in the analysis. Of these, 19.6% (n=132,120) were obese, and 80.3% (n=541,959) were non-obese. The mean age of obese and non-obese patients was 64.33 and 69.10 years, respectively. 62.91% of the obese and 56.07% of the non-obese patients were females. Caucasians (77.73 vs. 73.45%) and Pacific Islanders (1.29% vs. 0.66%) were more prevalent in the non-obese group, whereas African Americans (13.95 vs. 17.87%) and Hispanics (4.73% vs. 5.74%) tended to be more prevalent in the obese group. Type 2 diabetes mellitus, chronic kidney disease, atrial fibrillation, and pulmonary hypertension were significantly more prevalent among obese patients, while hypertension and smoking were more prevalent in the non-obese group. Among the obese group, 40.65% (n=74485) were morbidly obese and made up the morbidly obese subgroup. The detailed baseline characteristics for the included patients are summarized in Table 1. 

**Table 1:** Patient characteristics and comorbidities

Variable	Non-obese (%)	Obese (%)	P-value
Total	80.3% (N=541959)	19.6 (N=132120)	
Female	56.07	62.91	<0.0001
Age (mean)	69.1 years	64.33 years	<0.0001
Race			<0.0001
Caucasians	77.73	73.45	
African americans	13.95	17.87	
Hispanics	4.73	5.74	
Pacific Islanders	1.29	0.66	
Native americans	0.56	0.59	
Others	1.74	1.69	
Charleston comorbity index (CCI)			<0.0001
CCI=1	33.52	17.88	
CCI=2	24.1	25.72	
CCI > or = 3	42.39	56.4	
Income quartile (median household income of the patients ZIP Code)			<0.0001
1-47,999$	38	39.12	
48,000-60,999$	27.73	28.13	
61,000-81,999$	20.97	21.02	
82,000$ +	13.3	11.74	
Insurance			<0.0001
Medicare	72.23	65.51	
Medicaid	13.67	18.88	
Private insurance	11.39	12.71	
Self pay	2.71	2.9	
Type 2 Diabetes mellitus	11.5	19.12	<0.0001
Essential Hypertension	37.92	32.2	<0.0001
End stage renal disease	23.5	22.3	0.2527
Chronic kidney disease	13.23	17.93	<0.0001
Atrial Fibrillation	19.89	22.22	<0.0001
Smoking	42.23	40.32	<0.0001
Pulmonary Hypertension	1.58	2.8	<0.0001

Clinical and hospital-related outcomes in non-obese, obese, and morbidly obese patients admitted for COPD exacerbation. 

Among patients hospitalized for COPD exacerbations, mortality rates were lower among obese patients (2.1% vs. 2.87%) with an adjusted odds ratio of 0.72 [0.65, 0.80], p < 0.0001. Mortality rates were also lower (aOR, 0.88 [0.77-0.99], p = 0.03) among patients with morbid obesity (BMI > 40 kg/m^2^) when compared to those with a BMI < 40 kg/m^2^. 

Non-invasive ventilation was required in 12.16% of non-obese and 19.7% of obese patients. Mechanical ventilation was required in 7.5% of non-obese and 11.27% of obese patients. Obese patients were more likely to require non-invasive ventilation (aOR 1.63 [1.55, 1.7], p<0.0001) and mechanical ventilation (aOR 1.25 [1.19, 1.31], p<0.0001). The odds of requiring NIV (23.3%) and MV (13.85%) were even higher among morbidly obese patients at aOR 1.95 [1.85-2.05], p<0.0001, and aOR 1.53 [1.44-1.62], p<0.0001, respectively.

The primary outcomes, like mortality and the incidence of mechanical and non-invasive ventilation in the obese and morbidly obese subgroups, are summarized in Table 2.

**Table 2:** Primary outcomes in obese patients

Variable	Non obese (%)	Obese (%)	Odds Ratio (OR)	p-value	Adjusted OR	p-value
Mortality	2.87 (N= 15554)	2.1 (N=775)	0.726	<0.0001	0.72 [0.65, 0.80]	<0.0001
Non Invasive ventilation	12.16	19.7	1.77	<0.0001	1.63 [1.55,1.7]	<0.0001
Mechanical ventilation	7.52	11.27	1.56	<0.0001	1.25 [1.19, 1.31]	<0.0001

The primary outcomes, like mortality and the incidence of mechanical and non-invasive ventilation in the morbidly obese subgroup, are summarized in Table 3. 

**Table 3:** Primary outcomes in the morbidly obese subgroup of patients

Variable	BMI<40 (%)	Morbid obesity (%)	Odds Ratio (OR)	p-value	Adjusted OR	p-value
Mortality	2.87 (N= 15554)	2.34 (N=1662)	0.844	0.004	0.88 [0.77, 0.99]	0.043
Non Invasive Ventilation	12.48	23.3	2.13	<0.0001	1.95 [1.85,2.05]	<0.0001
Mechanical ventilation	7.59	13.85	1.95	<0.0001	1.53 [1.44, 1.62]	<0.0001

The rate of tracheostomy was 1.17%, 0.83%, and 0.38% among patients with morbid obesity, obesity, and BMI < 30 mg/kg with higher odds among obese (aOR 1.7 [1.42-2.04], p<0.0001) and morbidly obese patients (aOR 2.25 [1.85-2.75], p<0.0001). Obese and morbidly obese COPD patients were more likely to be discharged to a skilled nursing facility, with aOR 1.32[1.27-1.38] and aOR 1.37 [1.3-1.43], respectively. The odds of developing a cardiac arrest among obese vs. non-obese patients (aOR 0.89, [0.78, 1.02] p=0.09) were comparable. There was no increased risk of cardiac arrest among morbidly obese patients as well (aOR 1.05, [0.89, 1.24], p = 0.5). The odds of developing septic shock were comparable among non-obese, obese (p=0.14), and morbidly obese patients (p =0.05). Obese (aOR 1.11 [1.07-1.14], p<0.0001) and morbidly obese patients (aOR 1.21 [1.16-1.26], p<0.0001) had higher odds of being discharged on home oxygen as compared to non-obese patients. 

Average hospital charges were highest for morbidly obese patients ($59776), high in obese patients ($54849), and lowest for non-obese patients ($44958), p < 0.0001. Similarly, the mean length of stay was highest among morbidly obese patients (5.61 days), high among obese patients (5.22 days), and lowest among non-obese patients (4.51 days), and this difference was statistically significant (p < 0.0001). 

The secondary outcomes like the rate of tracheostomy, the incidence of adverse events like cardiac arrest and septic shock, discharge with supplemental oxygen, discharge to a skilled nursing facility, average hospital cost, and length of stay in the obese and morbidly obese subgroup are summarized in Table 4.

**Table 4:** Secondary outcomes in the obese patients.

Variable	Non obese	Obese	Odds Ratio (OR)	p-value	Adjusted OR	p-value
Tracheostomy	0.38	0.83	2.2	<0.0001	1.7 [1.42, 2.04]	<0.0001
Supplemental oxygen on discharge	30.53	32.32	1.08 [1.05, 1.12]	<0.0001	1.11 [1.07, 1.14]	<0.0001
Skilled Nursing Facility	14.51	16.47	1.15 [1.1, 1.2]	<0.0001	1.32 [1.27, 1.38]	<0.0001
Cardiac arrest	1.1	1.19	1.08	0.2	0.89 [0.78, 1.02]	0.093
Septic shock	0.55	0.58	1.07[0.9,1.27]	0.44	0.87 [0.71, 1.04]	0.14
Length of stay	4.51 days	5.22 days				<0.0001
Charges	$44958	$54849				<0.0001

 The secondary outcomes in the morbidly obese subgroup are summarized in Table 5.

**Table 5:** Secondary outcomes in the morbidly obese sub-group

Variable	BMI<40	Morbid obesity	Odds Ratio (OR)	p-value	Adjusted OR	p-value
Tracheostomy	0.38	1.16	3.05	<0.0001	2.25 [1.85, 2.75]	<0.0001
Supplemental oxygen on discharge	30.51	33.98	1.17 [1.13, 1.21]	<0.0001	1.21 [1.16, 1.26]	<0.0001
Skilled nursing facility	14.5	18.86	1.37 [1.3, 1.43]	<0.0001	1.73 [1.64, 1.82]	<0.0001
Cardiac arrest	10.8	13.8	1.27 [1.09,1.48]	0.001	1.05 [0.89, 1.24]	0.5
Septic shock	0.54	0.66	1.21 [0.98, 1.5]	0.066	0.95 [0.75, 1.19]	0.65
Length of stay	4.54 days	5.61 days				<0.0001
Charges	$44958	$59776				<0.0001

## Discussion

Obesity is a worldwide concern and has a significant impact on the morbidity and mortality associated with most chronic illnesses, including COPD [12,13]. This study analyzed the largest available US clinical registry from the year 2019, which included over 670,000 COPD-related hospitalizations. The key findings from our contemporary analysis of the NIS are as follows 1) Among COPD-related hospitalizations, obese patients had lower odds of mortality as compared to non-obese patients, 2) Odds of requiring NIV, MV, Tracheostomy were higher among obese patients as compared to non-obese patients admitted for COPD exacerbations. 3) Obese and morbidly obese patients had higher LOS and hospital charges when admitted for COPD exacerbations. 4) Odds of undergoing tracheostomy or requiring supplemental oxygen at discharge were higher among obese patients admitted for AECOPD. We observed that obese COPD patients were younger than the non-obese patients suggesting an earlier onset of morbidity among this group. Females were more prevalent in the obese group, which is consistent with the higher prevalence of obesity in females [14].

We found that mortality rates were lower in obese and morbidly obese patients as compared to nonobese patients. The results of our study were consistent with what has been reported before [10,15]. There have been reports of obese or overweight individuals having favorable survival outcomes. This was hypothesized as being secondary to better-preserved lung function, muscle mass, and exercise tolerance and not from fat accumulation [16]. Another hypothesis states that advanced COPD itself may lead to weight loss among obese patients; thus, patients are thought to be ‘non-obese’ at the time of death. Thus COPD-related mortality may appear more in patients with a normal BMI [9]. The obesity paradox is well documented, as seen in a large study that looks at 180,000 admissions for COPD across multiple states [17-20]. 

In our study, we assessed in-hospital morbidity represented by the need for mechanical ventilation and non-invasive ventilation. We observed that the odds of requiring both MV and NIV were significantly higher in the obese and morbid groups. Obese individuals had almost twice the odds of needing intubation and mechanical ventilation. NIV has also been increasingly reported to be responsible for favorable outcomes in all COPD patients regardless of BMI [21,22]. The higher rates of NIV and MV in COPD among obese individuals could be partly attributed to the co-existence of other pulmonary comorbidities, such as obstructive sleep apnea or obesity hypoventilation syndrome [23].

The average length of stay was higher among the obese and morbidly obese patients, which is congruent with the increased use of non-invasive and invasive ventilation in these groups. The impact of NIV and mechanical ventilation on a longer length of stay has been reported before [24,25]. This reflects higher morbidity when compared with non-obese individuals. Costs of care increased as the severity of obesity increased among these patients, with morbidly obese patients having the most amount of burden on healthcare utilization and non-obese patients having the least. 

Clinical adverse events such as cardiac arrest and septic shock were similar among obese and non-obese patients. The incidence of tracheostomy was higher in the obese group and almost twice as higher in the morbidly obese group, which suggests a presumed longer duration of recovery and morbidity in these groups [26]. Obesity was found to have an increased tendency to require supplemental oxygen, also suggesting a higher morbidity status and disease severity in this group [27,28].

Our study focuses primarily on investigating the effects of obesity on the morbidity of COPD and is the first of its kind to our knowledge. The use of NIV and MV are indicators of in-hospital morbidity, and length of stay and hospital charges represent healthcare resource utilization. The incidence of tracheostomy and discharge with supplemental oxygen are parameters of post-discharge morbidity, which suggests that such patients if discharged to rehabilitation centers or nursing homes, could further stretch out health care resources. Our study has several strengths; the NIS is one of the largest databases, and thus, the statistical analyses have high power. 

We acknowledge that our study also has some inherent limitations. The ICD 10 codes are primarily for billing purposes, so the sensitivity of the clinical information is limited. The parameters of obesity have been established using the secondary diagnosis of obesity; hence, it is difficult to ascertain the degree of obesity within the range. NIS data records hospitalizations and not individuals; hence, an individual with multiple admissions can be counted as multiple encounters. The data does not represent a linear timeline, and the secondary diagnoses could precede or follow the primary reason for hospitalization. NIS studies can only establish association without any comment on causation. We did not include the underweight category. 

## Conclusions

The presence and degree of obesity affect morbidity, as evidenced by higher rates of NIV, mechanical ventilation, tracheostomy, and discharge with supplemental oxygen among obese individuals admitted for COPD exacerbation. While obesity has been implicated in worse outcomes in many disease processes, it has not been associated with higher mortality in COPD patients. Obese COPD patients have a higher length of stay and hospital charges that suggest that they have a greater burden on the health care system.

## Appendices

List of ICD 10 codes

Table 6 shows the list of the ICD 10 codes used.

**Table 6:** List of ICD- 10 codes used

ICD 10 codes used	
COPD	J41, J41.0, J41.1, J41.8, J42, J43, J43.1, J43.2, J43.8, J44.0, J44.9, J43.9, J44.1
Acute respiratory failute	J96.00, J96.01, J96.02, J96.91, J80, R06.03, J96.21, J96.22, J96.20, J86.22, J96.21, R09.2
Obesity	E66.0, E66.1, E66.2, E66.8, E66.9, E66.01, E66.09, Z68.3, Z68.4
Morbid obesity	E66.0, E66.1
Non Invasive Ventilation	5A09357, 5A09358, 5A09557, 5A09457, 5A09458, 5A09558
Mechanical Ventilation	0BH18EZ, 0BH17EZ, 5A1935Z, 5A1945Z, 5A1955Z
Type 2 diabetes mellitus	E111, E112, E113, E114, E115, E116, E117, E118, E119
Hypertension	E10, E11
End stage renal disease	I12.0, I13.11, I13.2, N18.6
Chronic kidney disease	N18, N18.1, N18.2, N18.3, N18.30, N18.31, N18.32, N18.4, N18.5, N18.6
Atrial fibrillation	I48, I48.0, I48.11, I48.19, I48.2, I48.20, I48.21, I48.9, I48.91
Smoking history	Z87.89.1, F17.200
Pulmonary hypertension	I27.0, I27.2, I27.21, I27.22, I27.23, I27.24, I27.29
Oxygen dependence	Z9981
Tracheostomy	0B110F4, 0B110Z4, 0B113F4, 0B113Z4, 0B114F4, 0B114Z4
Cardiac arrest	I46, I46.2, I46.8, I46.9
septic shock	R65.21
